# Quality of internet-based decision aids for shoulder arthritis: what are patients reading?

**DOI:** 10.1186/s12891-018-2018-6

**Published:** 2018-04-11

**Authors:** Jeremy S. Somerson, Aaron J. Bois, Jeffrey Jeng, Kamal I. Bohsali, John W. Hinchey, Michael A. Wirth

**Affiliations:** 1 University of Texas Medical Branch, 301 University Blvd, Galveston, TX 77555 USA; 2 Sport Medicine Centre, University of Calgary, 2500 University Drive NW, Calgary, AB T2N 1N4 Canada; 3 University of California Los Angeles, Los Angeles, USA; 4 Jacksonville Orthopaedic Institute-Beaches Division, 6100 Kennerly Road Suite 101, Jacksonville, FL 32216 USA; 5 Department of Orthopaedics, The University of Texas Health Science Center San Antonio, 7703 Floyd Curl Drive – MC 7774, San Antonio, TX 78229 USA

**Keywords:** Shoulder, Arthritis, Internet-based information

## Abstract

**Background:**

The objective of this study was to assess the source, quality, accuracy, and completeness of Internet-based information for shoulder arthritis.

**Methods:**

A web search was performed using three common Internet search engines and the top 50 sites from each search were analyzed. Information sources were categorized into academic, commercial, non-profit, and physician sites. Information quality was measured using the Health On the Net (HON) Foundation principles, content accuracy by counting factual errors and completeness using a custom template.

**Results:**

After removal of duplicates and sites that did not provide an overview of shoulder arthritis, 49 websites remained for analysis. The majority of sites were from commercial (*n* = 16, 33%) and physician (*n* = 16, 33%) sources. An additional 12 sites (24%) were from an academic institution and five sites (10%) were from a non-profit organization. Commercial sites had the highest number of errors, with a five-fold likelihood of containing an error compared to an academic site. Non-profit sites had the highest HON scores, with an average of 9.6 points on a 16-point scale. The completeness score was highest for academic sites, with an average score of 19.2 ± 6.7 (maximum score of 49 points); other information sources had lower scores (commercial, 15.2 ± 2.9; non-profit, 18.7 ± 6.8; physician, 16.6 ± 6.3).

**Conclusions:**

Patient information on the Internet regarding shoulder arthritis is of mixed accuracy, quality, and completeness. Surgeons should actively direct patients to higher-quality Internet sources.

## Background

Internet growth and popularity over the past two decades has required healthcare to evolve in many ways. Traditional methods of disseminating medical information (i.e., pamphlets from local healthcare providers) have been largely replaced by websites. This expansion of health information available on the Internet has continued at an accelerated pace. The search engine Google (www.google.com) yields over 21 million results when the search term “arthritis” is entered. The unregulated format of the Internet provides opportunity for any source to publish health-related information regardless of validity and veracity.

Recent surveys have found that nearly 81% of U.S. adults use the Internet and, of those, 72% reported searching for health-related information online [1]. Many patients choose the Internet as their initial source for information to evaluate medical conditions before deciding whether or not to seek a physician [1–4]. Consequently, the quality and informational content on the Internet has the capacity to substantially impact patient health outcomes.

Over the past decade, the orthopaedic community has started to evaluate the quality and accuracy of informational websites for specific diagnoses. A 2005 study found that the Internet-based content on scoliosis primarily originates from academic sites, yet is of limited quality and poor accuracy [5]. More recently, the results of a study evaluating anterior cruciate ligament reconstruction revealed progress in the quality of information compared to prior studies [6]; however, to the best of the authors’ knowledge, similar studies on shoulder arthritis have not been published.

Shoulder pain is a common orthopaedic complaint with a prevalence as high as 50% in the elderly population [7]. Shoulder arthritis is an increasing problem in the aging population and surgical treatment is becoming more common. A recent study in the United States found that nearly 47,000 shoulder arthroplasties were performed as inpatient procedures in 2008, which represented a 2.5-fold increase when compared to only 19,000 shoulder arthroplasties in 1998 [8].

The primary objective of this study was to assess the source, quality, accuracy, and completeness of Internet-based information for glenohumeral joint arthritis by objectively analyzing the content with a predetermined set of criteria. We hypothesized that the websites available for glenohumeral joint arthritis would be lacking in quality and incomplete in content. To our knowledge, a comprehensive assessment of Internet-based information for glenohumeral joint arthritis has not been previously performed.

## Methods

**Fig. 1:**
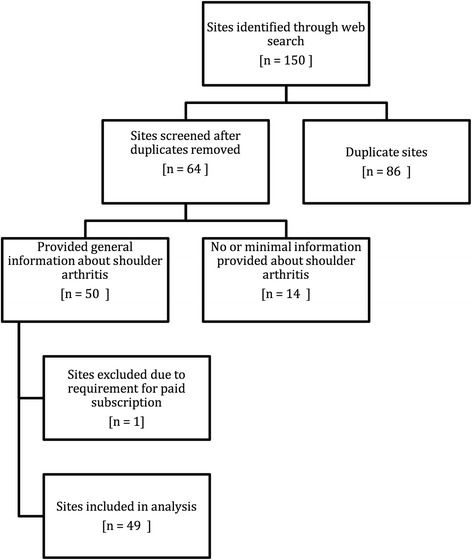
Flowchart demonstrating number of websites included and excluded at each stage of the review

To evaluate the quality and content of Internet information, the search term “shoulder arthritis” was entered into three major search engines (www.google.com, www.yahoo.com, and www.bing.com). These were chosen in an attempt to simulate real-world usage as research has shown that these three search engines account for over 89% of all Internet searches in the United States [9]. The first 50 websites from each search engine were selected to be included in our study. Paid advertising links were excluded from this study. Duplicate websites within and between search engines were excluded from the final list to ensure each website was only evaluated once. Also, links that did not relate to shoulder arthritis and websites requiring paid subscription were removed from the final list to more accurately simulate patient usage. Of the 150 potentially eligible websites to be evaluated by each reviewer, the final number of sites included after all exclusion criteria were applied was 49 (Fig. 1). Three independent orthopaedic surgeons with shoulder and elbow fellowship training graded the websites selected for this study (A.J.B., K.I.B. and J.W.H.).

A grading template was developed to evaluate the source, quality, accuracy and completeness of each website. Source was divided into 1 of 4 categories: 1) *academic* included any university-affiliated physician group; 2) *commercial* if any websites received industry funding, displayed advertisements, or sold products for profit; 3) *nonprofit* included any organization that does not earn profits for its owners; and 4) *physician* encompassed any professional sites for an individual or group of physicians not affiliated with an academic institution. In cases of website categorization discrepancy amongst reviewers, the final category was assigned based on the agreement between the 3 reviewers; however, if all 3 reviewers were in disagreement, the site in question was presented and reviewed as a group to reach consensus for final categorization.

The second aspect of the grading template evaluated the quality of information being presented on each site. The Health On the Net (HON) Foundation is a non-governmental organization serving to monitor and encourage the dissemination of quality healthcare information throughout the Internet. The HON Foundation developed a certification system for websites that choose to follow the HONcode principles established by the organization. Certified sites are permitted to display a HONcode seal of approval to demonstrate their commitment to providing reliable health and medical information on the Internet. Following the methods used by Starman et al. [10], each website’s quality of information was measured by a 16-point scale used to assess the compliance of websites according to the HON Foundation principles (HON Quality Score)(Table 1). If a site displayed a HONcode seal of approval, it was noted but still evaluated using the 16-point scale. A score of 0 indicated that none of the benchmarks for reliability and quality was met, while a score of 16 indicated that all benchmarks were met.

**Table 1:** Health On the Net Foundation (HON) Quality Score (as described by Starman et al. [10])

Transparency and honesty
• Transparency of provider of site – including name (1), physical address or electronic address (1) of the person or organization responsible for the site (2 points).
• Transparency of purpose and objective (1) of the site (1 point).
• Target audience (1) clearly defined (further detail on purpose, multiple audiences could be defined at different levels)(1 point).
• Transparency of all sources of funding (1) for site (grants, sponsors, advertisers, nonprofit, voluntary assistance)(1 point).
Authority
• Clear statement of sources for all information (0 = none, 1 = some, and 2 = all) provided and date of publication (1) of source (3 points).
• Names and credentials of all human/institutional authors of information (0 = none, 1 = some, and 2 = all) put up on the site, including dates at which credentials were received (2 points).
Privacy and data protection
• Privacy (1) and data protection policy and system for the processing of personal data, including processing invisible to users (1 point).
Updating of information
• Clear and regular updating of the site, with date of update clearly displayed for each page and/or item as relevant (1 = listed). Regular checking of relevance of information (1 point).
Accountability
• Accountability—user feedback (1), and appropriate oversight responsibility (such as a named quality compliance officer (1) for each site) (2 points).
• Responsible partnering—all efforts should be made to ensure that partnering or linking to other web sites is undertaken only with trustworthy individuals and organizations who themselves comply with relevant codes of good practice
• Editorial policy—clear statement describing what procedure was used for selection of content (1 point).
Accessibility
• Accessibility—attention to guidelines on physical accessibility as well as general findability, searchability, readability (1 = clear organization of topics without embedded advertisements, etc.), and usability (1 point).

Accuracy of the source in question was calculated by counting the number of factual errors identified by each reviewer. When disagreements occurred, the average number of errors identified for each site from the three reviewers was recorded. Content completeness was graded on a custom 49-point scale (Table 2) developed by two orthopaedic surgeons (J.S.S. and M.A.W.) based on a previously-described algorithm [5]. Criteria were generated for 4 categories of information through a review of information in 4 shoulder textbooks [11–14]: 1) disease pathophysiology, pathoanatomy, and pathogenesis; 2) clinical evaluation; 3) treatment; 4) indications, outcomes, and complications. Disease-specific concepts were calculated based on frequency of appearance in each textbook. The final grading rubric excluded any inconsistent and/or low-yield information. For completeness, a board-certified orthopaedic surgeon with shoulder and elbow fellowship training reviewed all of the grading criteria. Each website was awarded 1 point for every disease-specific concept mentioned, for a maximum score of 49 points.

**Table 2:** Shoulder Arthritis Content

Disease Pathophysiology, Pathoanatomy, and Pathogenesis	
- Mean score: 5.1±2.0 (maximum score 15)	
• Description of glenohumeral joint structures	
• Physical wear leading to articular cartilage failure and degeneration	
• Humeral head changes	
• Glenoid changes	
• Posterior humeral head subluxation	
• Anterior capsule contracture	
• Joint space narrowing	
• Age	
• Post-traumatic	
• Systemic factors (gender, smoking, diabetes, genetics)	
• History of previous surgery for glenohumeral instability	
• Increased load bearing	
• Cuff tear arthropathy	
• Avascular necrosis	
• Autoimmune disease	
Clinical Evaluation	
- Mean score 5.1±2.1 (maximum score 12)	
• Complaint of pain	
• History of disease	
• Sleep difficulties	
• Progression of functional difficulties	
• Joint crepitation	
• Limited glenohumeral motion/loss of external rotation	
• Tenderness over posterior joint line	
• Muscle weakness/atrophy	
• Plain radiographs	
• CT scan	
• Arthrography	
• MRI	
Treatment	
- Mean score 5.2±1.4 (maximum score 11)	
• Patient education	
• Rest/avoiding provocative activities (i.e., activity modification)	
• Physical therapy	
• Anti- inflammatory medications	
• Corticosteroid injection	
• Arthroscopy	
• Hemiarthroplasty	
• Total shoulder arthroplasty	
• Reverse total shoulder arthroplasty	
Indications, Outcomes, and Complications	
- Mean score: 1.5±1.8 (maximum score 11)	
• Pain/stiffness/weakness	
• Degree of dysfunction unacceptable to patient	
• Severe pain failing conservative (i.e., nonoperative) treatment	
• Expected outcomes and success rates of surgery	
• Rehabilitation protocols	
• Inherent surgical complications	
• Component loosening	
• Instability	
• Periprosthetic fractures	
• Rotator cuff tears	
• Neurologic injuries	

### Statistical analysis

Descriptive statistics were generated based on average scores for content score, HON quality score, and number of errors reported from each reviewer. Mean and standard deviation were reported for these 3 outcome measures by website authorship. These measures were then compared by authorship group of websites (academic, commercial, non-profit, physician) to all other websites using a non-parametric 2-sample Wilcoxon rank sum test. Statistical significance was established at *p* ≤ 0.05. Consistency between the 3 graders was measured using Cronbach’s alpha statistic for completeness and accuracy outcome measures.

## Results

The distribution of sites by authorship was as follows: 12 (24%) were considered to be from an academic source, 16 (33%) were commercial, 5 (10%) were from non-profit organizations, and 16 (33%) were considered physician-based.

**Fig. 2:**
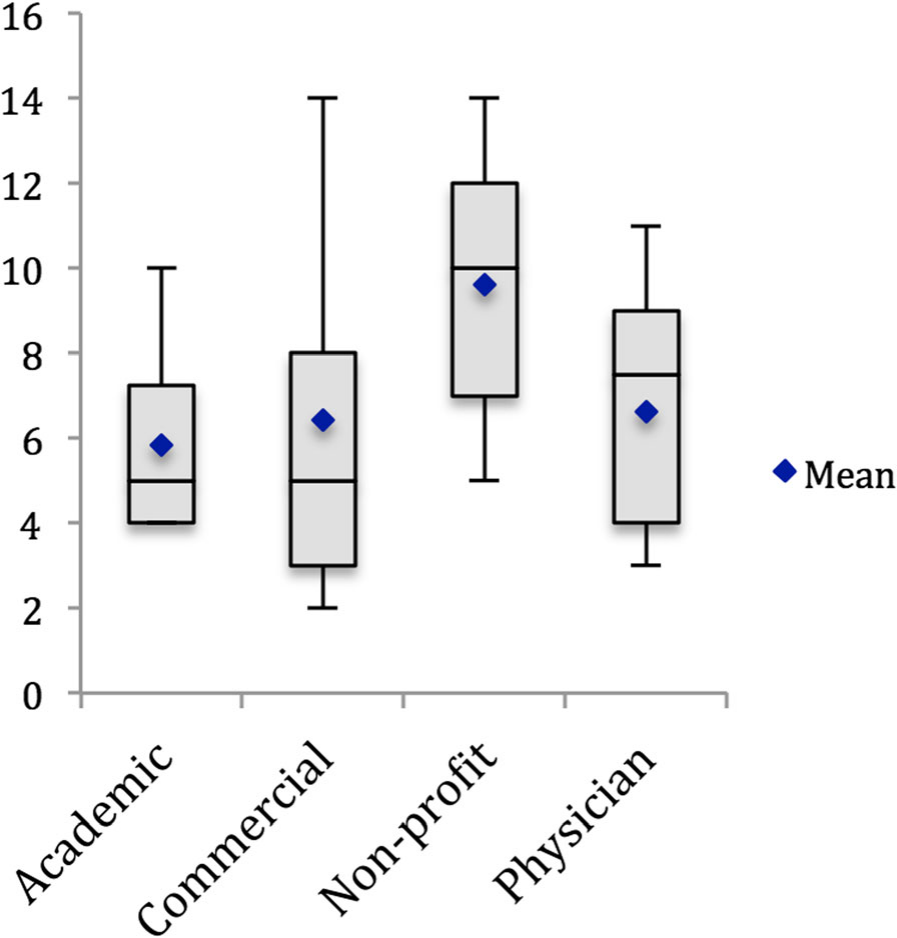
Box plot depicting the HON Quality Score by authorship. Bars represent maximum and minimum data values

**Fig. 3:**
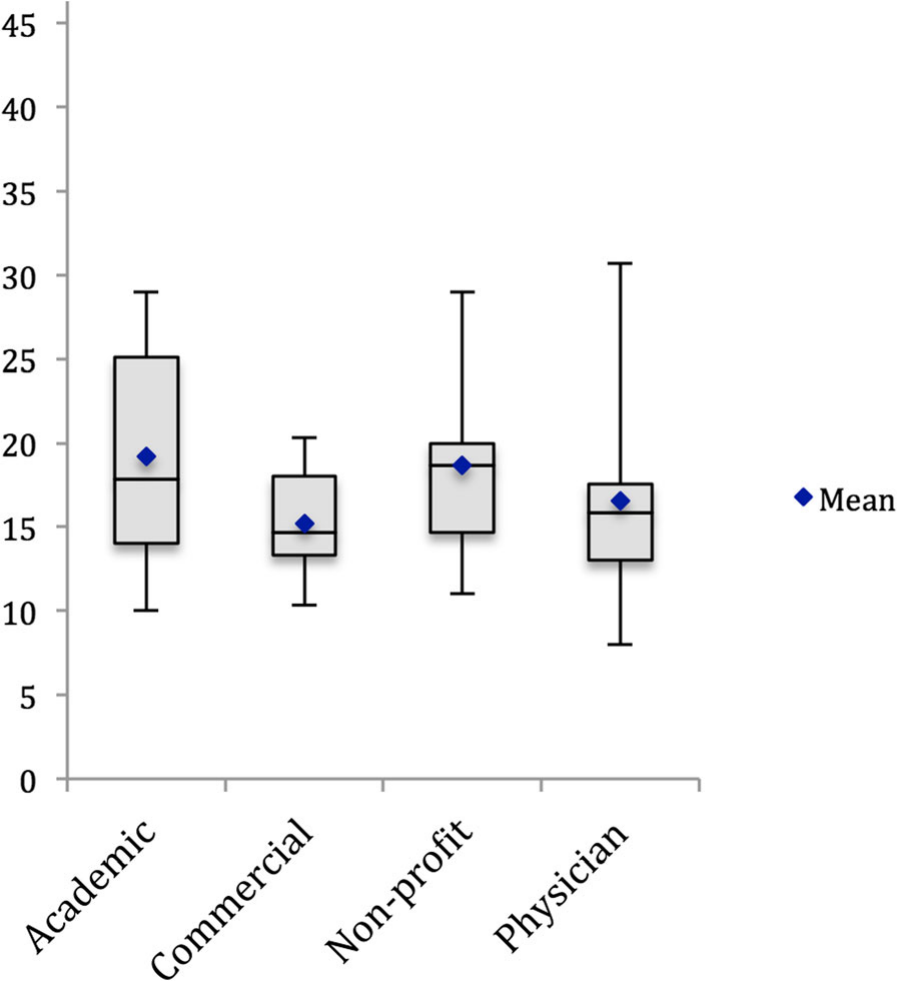
Box plot depicting content completeness score by authorship. Bars represent maximum and minimum data values

The HON Quality score was 5.8 ± 2.1 (mean ± SD) for academic sites, 6.4 ± 4.2 for commercial sites, 9.6 ± 3.6 for non-profit sites and 6.6 ± 2.7 for physician sites on a maximum 16-point scale (Fig. 2). We found only 3 out of 49 websites reviewed displayed the HONcode compliance seal of approval, but none of those websites received a full score using the 16-point scale. The top HON quality score of 14 points was attained by 4 websites (3 commercial and 1 non-profit website). The average number of mistakes (i.e., factual errors) per site was 0.2 (range: 0–1) for academic sites, 1.0 (range 0–3) for commercial sites, 0.5 (range 0–2) for non-profit sites and 0.4 (range: 0–2) for physician sites. These mistakes most commonly included unproven statements, such as the ability of shoulder injections or nutritional supplements to improve the health of articular cartilage. Non-profit sites had significantly higher HON quality scores than other types of sites (*P* < 0.05). Commercial sites had more errors than other types of sites (*P* < 0.01) while academic sites had fewer errors (*P* = 0.02). The content completeness score was 19.2 ± 6.7 (mean ± SD) for academic sites, 15.2 ± 2.9 for commercial sites, 18.7 ± 6.8 for non-profit sites and 16.6 ± 6.3 for physician sites on a maximum 49-point scale (Fig. 3). The 5 websites with the highest content completeness scores are presented in Table 3. We further analyzed each website’s content completeness score and found consistently low scores for the subcategory of “Indications, Outcomes, and Complications”; the average score was 1.5 out of 11 possible subcategory points (13.6%) (Table 1).

**Table 3:** Top-rated shoulder arthritis websites for content completeness

Site Name	Hyperlink	Content Score (49-point maximum)
The Steadman Clinic – Glenohumeral Arthritis	www.thesteadmanclinic.com/patient-education/shoulder/glenohumeral-arthritis	31 (Path: 9.7, Eval: 10.3, Tx: 6.0, I/O/C: 4.7)
Shoulder Arthritis and Rotator Cuff Tears: Causes of Shoulder Pain	shoulderarthritis.blogspot.com	30 (Path: 10.7, Eval: 6.7, Tx: 7.0, I/O/C: 6.0)
UCLA Health – Shoulder Arthritis	ortho.ucla.edu/body.cfm?id=181	29 (Path: 9.0, Eval: 6.7, Tx: 6.0, I/O/C: 7.3)
Arthritis of the Shoulder - OrthoInfo – AAOS	orthoinfo.aaos.org/en/diseases--conditions/arthritis-of-the-shoulder	29 (Path: 9.0, Eval: 7.3, Tx: 9.0, I/O/C: 3.7)
University of Washington - Shoulder Arthritis	http://www.orthop.washington.edu/?q=patient-care/shoulder-arthritis.html	29 (Path: 10.0, Eval: 9.3, Tx: 7.3, I/O/C: 2)

Path Pathology Content subscore, Eval Clinical Evaluation subscore, Tx Treatment subscore, I/O/C Indications/Outcomes/Complications subscore

### Interobserver reliability

Total scores for content completeness points on a 49-point scale demonstrated excellent interobserver item reliability (Cronbach’s alpha: 0.90). Subgroups for content completeness also revealed a high level of consistency (Disease Pathophysiology: 0.84, Clinical Evaluation: 0.89, Treatment: 0.86, Indications, Outcomes, and Complications: 0.86). The number of mistakes detected per website demonstrated good consistency (Cronbach’s alpha: 0.67).

## Discussion

Variability in the content that patients read on the Internet can present an obstacle to informed decision-making. This study reviewed Internet-based resources regarding shoulder arthritis and characterized the source, quality, accuracy and completeness of the information.

One of the first orthopaedic studies to look at Internet-based information evaluated the type, quality, and reliability of information regarding carpal tunnel syndrome [15]. The authors of this study found that only 23% of the 49 sites were created by a physician or an academic organization. Moreover, neither category scored above 40%, with an informational value score that ranged from 0 to 100. In 2005, Mathur et al. [5] conducted a similar study using 5 search engines and the key word “scoliosis,” and found that the majority of websites for scoliosis were academic; however, they determined the content and accuracy of those sites to be poor. They found that 21 out of the 50 websites evaluated had an accuracy score of less than 25%.

In our study, academic websites had the highest average content completeness score of 19.2 out of 49 (39.2%), whereas commercial websites had the lowest average content completeness score of 15.2 (31%). However, we found that certain popular commercial sites, such as Medscape and WebMD, had content completeness scores above the category’s average (47% and 41%, respectively). Physician authorship of individual content pages on these commercial sites may have contributed to this finding.

Regarding distribution of sources, we found commercial (33%) and physician-based (33%) websites were most common, followed closely by academic websites (24%) and lastly by non-profit websites (10%). There were no personal websites identified. Of the top 10 scores for content completeness, 5 were from academic organizations, 2 each from physician-based and non-profit groups, and only 1 from a commercial website. These results may reflect recent efforts by academic institutions and other healthcare organizations to provide patients with accurate and comprehensive healthcare information. Nevertheless, over 85% of all websites had content scores of less than 24 points, suggesting improvement is warranted. In particular, the low scores for the subcategory of “Indications, Outcomes, and Complications” is consistent with a previous orthopaedic study that found a lack of information on potential complications of various treatments [16].

The HON Foundation evaluates health-related sites and has established guidelines in an attempt to standardize the reliability and credibility of medical information on the Internet. Candidate websites are subjected to a thorough evaluation based upon HON ethical standards and, if certified, are permitted to display a HONcode seal of approval free of charge. The ethical aspects of the HONcode include the author’s credentials, the date of the last modification with respect to clinical documents, confidentiality of data, source data reference, funding, and advertising [17]. However, over 83% of websites scored below 10 points (of a maximum of 16 points) to suggest that the majority of glenohumeral arthritis websites are of relatively low quality. This is consistent with recent studies that have found low quality Internet information for both shoulder- and elbow-related disorders, such as shoulder instability [18], and rotator cuff tears [19], and ulnar collateral ligament injuries [20], as well as shoulder-related surgery, such as total shoulder arthroplasty [21].

Ideally, a high-quality patient information website should be developed by a credible source and display accurate information regarding diagnostic tests, treatment options, outcomes, and possible complications. Information should also be at a reading level that is comprehensible to an average patient, as recent studies have shown patient materials on the Internet to be written at a much higher level than many patients can comprehend [18–20, 22]. The OrthoInfo site (www.orthoinfo.aaos.org), created by the American Academy of Orthopaedic Surgeons (AAOS), scored among the top five websites for HON score and content completeness. The OrthoInfo site received 14 out of 16 points for HON score and 29 (59%) out of 49 points for content completeness. Patients should be actively directed to this and other high-scoring information sources.

There were a few limitations of this study that need to be considered. Although we used the 3 most popular Internet search engines, our method for selecting websites may not accurately reflect the way in which all patients locate a website for medical information (i.e., not all patients use Google, Yahoo, or Bing as their primary search engines). Second, selection of the top 50 websites may be affected by strategies for website optimization, as some sites may employ commercially available methods for improving their search engine ranking. Third, it was often unclear to what extent third parties were used to provide content for each website; this could have an impact on accuracy and completeness. Lastly, the subjective nature of the grading system created to evaluate each website is a potential source of bias; we did not engage patients or non-physician health care providers in the process to better understand what would constitute complete information from other perspectives. To limit the variability of the selected criteria, 2 orthopaedic surgeons developed the grading rubric through a systematic review of 4 primary shoulder textbooks. This resulted in a high level of interobserver reliability for total content completeness scores as well as subgroup scores.

## Conclusions

The majority of information on the Internet regarding shoulder arthritis is of mixed quality and comprehensive sources are lacking. One-third of the top-ranked websites are commercial in nature and were more likely to contain factual errors. Academic sites were less likely to contain factual errors, and non-profit sites met a greater number of quality measures for information sources. We believe orthopaedic surgeons should be knowledgeable about where patients are likely to browse for medical information on the Internet and be responsible in directing them to optimal sources.

## Abbreviations

AAOS: American Academy of Orthopaedic Surgeons
HON: Health On the Net

## References

[1] Fox S, Duggan M. Health Online 2013. Pew Internet and American Life Project. 2013. http://www.pewinternet.org/~/media//Files/Reports/PIP_HealthOnline.pdf. Accessed 2 Feb 2014.

[2] Benigeri M, Pluye P (2003). Shortcomings of health information on the internet. Health Promot Int.

[3] Diaz JA, Griffith RA, Ng JJ, Reinert SE, Friedmann PD, Moulton AW (2002). Patients’ use of the internet for medical information. J Gen Intern Med.

[4] Hesse BW, Nelson DE, Kreps GL, Croyle RT, Arora NK, Rimer BK (2005). Trust and sources of health information. Arch Intern Med.

[5] Mathur S, Shanti N, Brkaric M, Sood V, Kubeck J, Paulino C, Merola A (2005). Surfing for scoliosis: the quality of information available on the internet. Spine.

[6] Duncan IC, Kane PW, Lawson KA, Cohen SB, Ciccotti MG, Dodson CC (2013). Evaluation of information available on the internet regarding anterior cruciate ligament reconstruction. Arthroscopy.

[7] Wofford JL, Mansfield RJ, Watkins RS (2005). Patient characteristics and clinical management of patients with shoulder pain in U.S. primary care settings: secondary data analysis of the National Ambulatory Medical Care Survey. BMC Musculoskelet Disord.

[8] Kim SH, Wise BL, Zhang Y, Szabo RM (2011). Increasing incidence of shoulder arthroplasty in the United States. J Bone Joint Surg Am.

[9] Purcell K, Brenner J, Rainie L. Search Engine Use 2012. Pew Internet and American Life Project. 2012. https://www.eff.org/files/Pew%202012_0.pdf. Accessed 3 Feb 2014.

[10] Starman JS, Gettys FK, Capo JA, Fleischli JE, Norton HJ, Karunakar MA (2010). Quality and content of internet-based information for ten common Orthopaedic sports medicine diagnoses. J Bone Joint Surg Am.

[11] Gartsman GM. Shoulder arthroscopy. 2nd ed: W.B. Saunders Company; 2009.

[12] Iannotti JP, Williams GR, Miniaci A, Zuckerman JD. Disorders of the shoulder, reconstruction. 3rd ed: Lippincott Williams & Wilkins; 2013.

[13] Rockwood CA Jr, Matsen FA, Wirth MA, Lippitt SB, Fehringer EV, Sperling JW. The shoulder. 4th ed: Elsevier Health Sciences; 2009.

[14] Williams GR Jr, Yamaguchi K, Ramsey ML, Galatz LM. Shoulder and elbow arthroplasty. 1st ed: Lippincott Williams & Wilkins; 2004.

[15] Beredjiklian PK, Bozentka DJ, Steinberg DR, Bernstein J (2000). Evaluating the source and content of Orthopaedic information on the internet: the case of carpal tunnel syndrome. J Bone Joint Surg Am.

[16] Lutsky K, Bernstein J, Beredjiklian PK (2013). Quality of information on the internet about carpal tunnel syndrome: an update. Orthopedics.

[17] Boyer C, Selby M, Scherrer JR, Appel RD (1998). The health on the net code of conduct for medical and health websites. Comput Biol Med.

[18] Garcia GH, Taylor SA, Dy CJ, Christ A, Patel RM, Dines JS (2014). Online resources for shoulder instability: what are patients reading?. J Bone Joint Surg Am.

[19] Dalton DM, Kelly EG, Molony DC (2015). Availability of accessible and high-quality information on the internet for patients regarding the diagnosis and management of rotator cuff tears. J Shoulder Elb Surg.

[20] Johnson CC, Garcia GH, Liu JN, Stepan JG, Patel RM, Dines JS (2016). Internet resources for Tommy John injuries: what are patients reading?. J Shoulder Elb Surg.

[21] Matthews JR, Harrison CM, Hughes TM, Dezfuli B, Sheppard J (2016). Web page content and quality assessed for shoulder replacement. Am J Orthop (Belle Mead NJ).

[22] Shah AK, Yi PH, Stein A (2015). Readability of Orthopaedic oncology-related patient education materials available on the internet. J Am Acad Orthop Surg.

